# Exploring Chemical Information in PubChem

**DOI:** 10.1002/cpz1.217

**Published:** 2021-08-09

**Authors:** Sunghwan Kim

**Affiliations:** ^1^ National Center for Biotechnology Information, National Library of Medicine National Institutes of Health Bethesda Maryland

**Keywords:** cheminformatics, chemical structure search, drug discovery, molecular similarity, PubChem, public database

## Abstract

PubChem (https://pubchem.ncbi.nlm.nih.gov) is a public chemical database that serves scientific communities as well as the general public. This database collects chemical information from hundreds of data sources and organizes them into multiple data collections, including Substance, Compound, BioAssay, Protein, Gene, Pathway, and Patent. These collections are interlinked with each other, allowing users to discover related records in the various collections (e.g., drugs targeting a protein or genes modulated by a chemical). PubChem can be searched by keyword (e.g., a chemical, protein, or gene name) as well as by chemical structure. The input structure can be provided using popular line notations or drawn with the PubChem Sketcher. PubChem supports various types of structure searches, including identity search, 2‐D and 3‐D similarity searches, and substructure and superstructure searches. Results from multiple searches can be combined using Boolean operators (i.e., AND, OR, and NOT) to formulate complex queries. PubChem allows the user to quickly retrieve a list of records annotated with a particular classification or ontological term. This paper provides step‐by‐step instructions on how to explore PubChem data with examples of commonly requested tasks. © 2021. This article is a U.S. Government work and is in the public domain in the USA. Current Protocols published by Wiley Periodicals LLC.

**Basic Protocol 1**: Finding genes and proteins that interact with a given compound

**Basic Protocol 2**: Finding drug‐like compounds similar to a query compound through a two‐dimensional (2‐D) similarity search

**Basic Protocol 3**: Finding compounds similar to a query compound through a three‐dimensional (3‐D) similarity search

**Support Protocol**: Computing similarity scores between compounds

**Basic Protocol 4**: Getting the bioactivity data for the hit compounds from substructure search

**Basic Protocol 5**: Finding drugs that target a particular gene

**Basic Protocol 6**: Getting bioactivity data of all chemicals tested against a protein.

**Basic Protocol 7**: Finding compounds annotated with classifications or ontological terms

**Basic Protocol 8**: Finding stereoisomers and isotopomers of a compound through identity search

## INTRODUCTION

PubChem (https://pubchem.ncbi.nlm.nih.gov; Kim, 2016; Kim et al., 2019; Kim et al., 2021; Kim et al., 2016) is a public chemical database created by the National Library of Medicine (NLM), an institute within the U.S. National Institutes of Health (NIH). With millions of unique users every month, PubChem is a very popular chemistry information resource for biomedical research communities in many areas, including cheminformatics, chemical biology, medicinal chemistry, and drug discovery. Importantly, PubChem also serves as a source of big data in chemistry, used in many machine learning and data science projects for virtual screening, computational toxicology, drug repurposing, etc.

PubChem's information content, collected from hundreds of data sources, is organized into multiple data collections, including Substance, Compound, BioAssay, Gene, Protein, Pathway, and Patent (Kim et al., 2021). Substance archives the chemical data submitted by individual data sources and Compound stores the unique chemical structures extracted from Substance through chemical structure standardization (Hähnke, Kim, & Bolton, 2018; Kim et al., 2016). BioAssay contains biological assay descriptions and test results deposited by assay data providers. The record identifiers (IDs) used in Substance, Compound, and BioAssay are called Substance ID (SID), Compound ID (CID), and Assay ID (AID), respectively. The other data collections (i.e., Gene, Protein, Pathway, and Patent) provide alternative views of PubChem data, related to a specific gene, protein, pathway, and patent document, respectively. Each record in the data collections has a dedicated web page (called a Summary page), which presents information available in PubChem for that record. This page also presents relevant annotations collected by PubChem from authoritative data sources.

PubChem's search interface, available on the PubChem homepage (https://pubchem.ncbi.nlm.nih.gov), allows users to simultaneously search the data collections using a text query. A chemical structure query can be used to perform various types of chemical structure searches, including identity, two‐dimensional (2‐D) and three‐dimensional (3‐D) similarity, and substructure and superstructure searches. In addition, PubChem provides various tools and services that help users to exploit PubChem data, which are described in detail in previous papers (Kim et al., 2019; Kim et al., 2021; Kim et al., 2016).

This article provides step‐by‐step instructions on how to perform common tasks in PubChem. In Basic Protocol 1, losartan (an antihypertensive drug) is used as an example to explain how to search PubChem by chemical name and find genes and proteins that interact with that chemical. Basic Protocols 2 and 3 focus on 2‐D and 3‐D similarity searches, respectively, which are described in detail in Background Information. Basic Protocol 2 shows how to find compounds structurally similar to losartan based on 2‐D similarity and how to filter them based on molecular properties to identify drug‐like compounds. Basic Protocol 3 demonstrates how to find compounds similar to losartan in terms of 3‐D similarity. In the Support Protocol, similarity scores between compounds are computed using the PubChem Score Matrix Service. In Basic Protocol 4, a substructure search is performed to identify compounds that share a common scaffold with losartan, and their bioactivity data is downloaded. Basic Protocol 5 shows how to search drugs that target a particular gene, and Basic Protocol 6 explains how to retrieve the bioactivity data for compounds tested against a given protein. In Basic Protocol 7, the PubChem Classification Browser is used to find compounds annotated with a classification or ontological term (e.g., antihypertensive agents). Finally, Basic Protocol 8 details how to perform an identity search to find stereoisomers and isotopomers of a given compound, using valsartan as an example.

## Basic Protocol 1 FINDING GENES AND PROTEINS THAT INTERACT WITH A GIVEN COMPOUND

The most common use of PubChem is to search for a specific piece of information on a chemical. This is typically done by performing a text search with a chemical name as a query, going to the Summary page of the best hit compound returned from the search, and locating the desired information on that page. This process is shown in Basic Protocol 1, which demonstrates how to find proteins and genes known to interact with losartan (CID 3961), a widely used antihypertensive drug.

The chemical‐protein and chemical‐gene interaction data in PubChem originate from multiple sources, such as DrugBank (Wishart et al., 2018), Comparative Toxicogenomics Database (CTD; Davis et al., 2021), Drug‐Gene Interaction Database (DGIdb; Freshour et al., 2021), IUPHAR/BPS Guide to PHARMACOLOGY (Armstrong et al., 2020), ChEMBL (Mendez et al., 2019), and RCSB Protein Data Bank (PDB; Burley et al., 2019). The biological test results for a chemical can also be a good source for its interactions with macromolecules. While the interaction data from DrugBank is retrieved in Basic Protocol 1 as an example, the chemical‐macromolecule associations from one data source are not necessarily the same as those from other sources. Therefore, it is recommended to access the data from all relevant sources and review the variances in the related records.

### Materials

An up‐to‐date Web browser, such as Google Chrome, Microsoft Edge, Safari, or Firefox, is required for this protocol (and all other protocols in this article)

**Figure 1:**
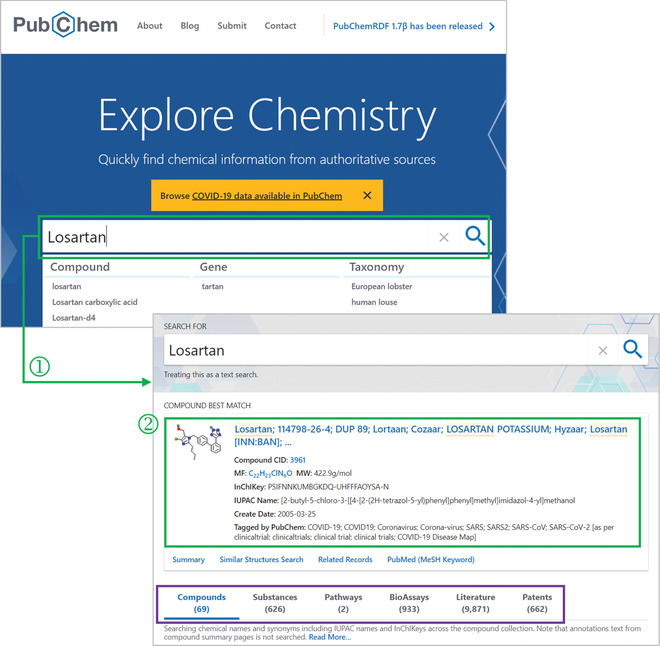
Searching PubChem using a text query. When a text query is provided (1), PubChem searches multiple collections for relevant records, and the hits from each collection can be viewed by clicking the corresponding tab (indicated in the purple box). When possible, PubChem suggests the best hit at the top of the search results. For example, when the chemical name losartan is used as a query, PubChem suggests CID 3961 as the best hit. Clicking this record or one of the hits found in the Compound collection directs the user to its compound page (2).

1 Go to the PubChem homepage (https://pubchem.ncbi.nlm.nih.gov).
  The PubChem homepage serves as the entry point for various PubChem services. It has a search box that accepts various types of text queries, and examples are provided below the search box. These examples include chemical names (e.g., aspirin), gene symbols (e.g., EGFR), chemical abstract services (CAS) registry numbers (e.g., 57‐27‐2), and molecular formulas (e.g., C9H8O4). It is also possible to search using line notations for chemical structures, such as the Simplified Molecular Input Line Entry System (SMILES; Weininger, 1988, 1990; Weininger, Weininger, & Weininger, 1989) and the IUPAC International Chemical Identifier (InChI; Heller, McNaught, Pletnev, Stein, & Tchekhovskoi, 2015). The integer‐type identifiers for PubChem records (i.e., SID, CID, and AID) can also be used as a query.
2 Type losartan in the search box and click on the search (magnifying glass) button (‘1’ in Fig. 1).
  PubChem's search interface has an autocomplete/autosuggestion function. When a query is typed in the search box, the interface suggests a list of potential queries. This allows the user to quickly search PubChem by clicking one of the suggested terms.

**Figure 2:**
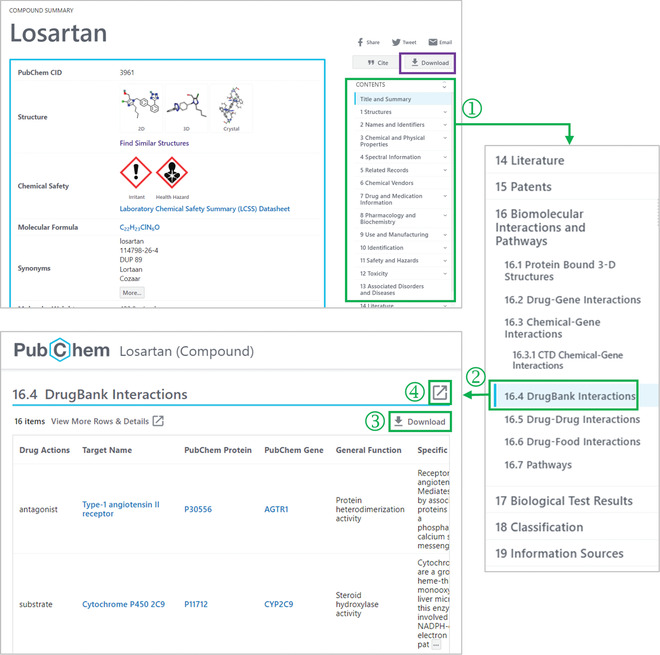
Navigating the Compound Summary page of losartan (CID 3961) (https://pubchem.ncbi.nlm.nih.gov/compound/3961). The user can navigate the Compound Summary page using the Table of Contents (1), available in the right column. One may find the macromolecules that losartan interacts with by clicking the “DrugBank Interactions” (2) from the Table of Contents. The information presented in each section can be downloaded by clicking the “Download” button (3). When there is too much information to present in a section of the Summary page, only the first few pieces of information are shown. To view all information available for the section, the user should click the full‐screen view button (4). All information presented on the Compound Summary page can be downloaded through the “Download” button available at the top‐right corner of the Compound Summary page (indicated in the purple box).

3 Click the best match shown at the top of the search results (‘2’ in Fig. 1) to go to the Summary page for the selected compound.
  When a search term is entered, PubChem simultaneously searches multiple data collections. The search result page has tabs that allow the user to view the hits from different collections (indicated in the purple box in Fig. 1). For compounds and substances (PubChem, 2014), a text query finds chemicals whose names match it. For other data collections (such as genes, proteins, pathways, literature, and patents), returned hits contain the query string within the records.
  When possible, PubChem tries to identify the most relevant record and display it at the top of the search result list. For the query losartan, PubChem identifies CID 3961 as the most relevant record. Clicking on this record directs the user to the Compound Summary page for CID 3961.
4 Go to the “DrugBank Interactions” subsection under the “Biomolecular Interactions and Pathways” section (‘2’ in Fig. 2), using the Table of Contents on the right column (‘1’ in Fig. 2).
  The Compound Summary page often contains a large amount of information, especially for well‐studied and well‐known compounds. The user can navigate this page using the Table of Contents, available in the right column. Alternatively, one may quickly search for a term or a particular string within the Summary page by pressing Ctrl+F (on a Windows/Linux PC) or Command+F (on a Mac) on the keyboard.
  The “DrugBank Interactions” subsection contains information on the macromolecules that interact with CID 3961 (losartan), curated by DrugBank. In DrugBank, macromolecules are classified into four groups according to the type of interaction with a drug molecule: targets, enzymes, carriers, and transporters. Among the macromolecules listed in this section, the “type‐1 angiotensin II receptor” is classified as a target, meaning that the therapeutic effect of losartan comes from its interaction with this protein.
  Clicking the target name “type‐1 angiotensin II receptor” in this section directs the user to the corresponding page in DrugBank, where more detailed information can be found. Additional information is available on the Gene and Protein Summary pages, which can be accessed by clicking the gene symbol (e.g., “AGTR1”) in the “PubChem Gene” column and the accession (e.g., “P30556”) in the “PubChem Protein” column. The PubChem Gene and Protein pages are further explained in Basic Protocols 5 and 6.
  Each section/subsection in a Summary page can be bookmarked for quick access. For example, the DrugBank Interactions subsection for CID 3961 is directly accessible via the URL https://pubchem.ncbi.nlm.nih.gov/compound/3961#section=DrugBank‐Interactions.

5 Click the “Download” button to download the list of the macromolecules interacting with losartan (‘3’ in Fig. 2).
  The data on the Compound Summary pages is regularly updated. When the data in an original data source is updated, this change is also reflected in PubChem through the next update cycle. Therefore, it is highly recommended to save the necessary data on a local computer.
6 If necessary, click the full‐screen view button (‘4’ in Fig. 2) to view all rows and columns.
  By default, a Summary page often shows only a part of the available data. For example, the “DrugBank Interactions” subsection in this protocol displays only the first few rows and columns of the tabular data. The remaining data can be viewed in the full‐screen view mode.

## Basic Protocol 2 FINDING DRUG‐LIKE COMPOUNDS SIMILAR TO A QUERY COMPOUND THROUGH 2‐D SIMILARITY SEARCH

PubChem's search interface provides many features beyond the simple text search. For example, it supports a search by chemical structure. A chemical structure can be used as a query for various types of structure searches, including identity search, 2‐D and 3‐D similarity searches, and substructure and superstructure searches. The input chemical structure can be specified with a line notation (e.g., SMILES or InChI) or drawn using the PubChem Sketcher. If the input structure already exists in the PubChem Compound database, its CID can be used as a query. It is also possible to initiate a chemical structure search from one of the hit compounds returned from a previous search. More details about chemical structure searches in PubChem are outlined in the Background Information section of this article.

Another important feature of PubChem's search interface is that it provides filters that limit the search results to only those records with the desired attributes. Each data collection has a different set of filters. For example, the compound records can be filtered based on several molecular properties, such as molecular weight, hydrogen bond donor and acceptor counts, rotatable bond count, etc. The assay records can be filtered based on data sources and assay types (e.g., in vivo, in vitro, cell‐based, biochemical, etc.). Taxonomy information can be used to filter the gene and protein records.

Basic Protocol 2, designed to demonstrate these two features (i.e., chemical structure search and filtering), aims to find drug‐like chemicals that are structurally similar to a given chemical. In this protocol, the CID of the best hit compound returned from the text query losartan (in Basic Protocol 1) is used to specify the input chemical structure for a subsequent 2‐D similarity search. The resulting compound list is further refined with filters to identify compounds that meet all criteria of Lipinski's rule of five (Lipinski, Lombardo, Dominy, & Feeney, 1997), which is a rule of thumb to evaluate drug‐likeness of molecules. The refined compound list, along with the computed properties, is downloaded on a local computer.

### Materials

An up‐to‐date Web browser, such as Google Chrome, Microsoft Edge, Safari, or Firefox, is required for this protocol (and all other protocols in this article)

**Figure 3:**
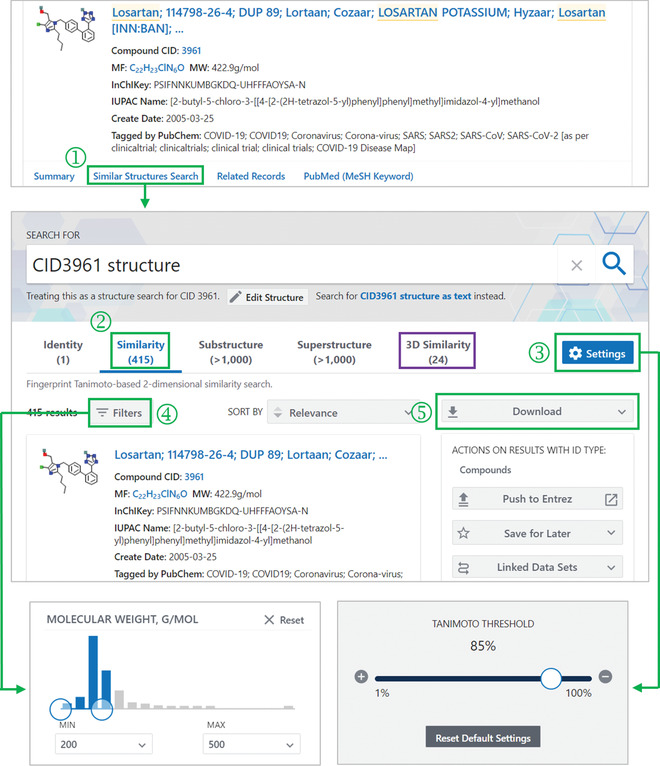
Performing a similarity search using a hit compound returned from a previous search. Each hit compound is presented with links that allow the user to access commonly requested data or services relevant to the compound. Among them is the “Similar Structures Search” link (1). Clicking this link will invoke multiple structure searches [including 2‐D similarity search (2)] using the compound as a query and present the search results. The user can rerun the 2‐D similarity search with a different similarity threshold (3) and apply filters (4) to refine the hit compounds based on several molecular properties. The hit compound list can be downloaded using the “Download” button (5). The result for the 3‐D similarity search can be viewed by clicking the “3D similarity” tab (indicated in the purple box).

1 Repeat steps 1‐2 of Basic Protocol 1 to search PubChem using losartan as a query.
2 Click the “Similar Structures Search” link at the bottom of the top panel that shows the best match (‘1’ in Fig. 3).
  For each hit compound returned from a search, PubChem provides links to the commonly requested information on that compound. One of the links is the "Similar Structures Search" link, which allows one to use that compound as a query to perform a 2‐D similarity search and other types of structure searches (i.e., identity, substructure, superstructure, and 3‐D similarity searches). As implied by the name of the link (“Similar Structures Search”), 2‐D similarity search results are shown by default (‘2’ in Fig. 3). The results of the other types of searches can be viewed by clicking the corresponding tab.

3 If necessary, click the Settings button (‘3’ in Fig. 3) and adjust the similarity threshold to a desired value.
  During a 2‐D similarity search, the similarity between the query and all compounds in PubChem is evaluated using the PubChem substructure fingerprint (PubChem, 2009) and the Tanimoto Coefficient (Chen & Reynolds, 2002; Holliday, Hu, & Willett, 2002; Holliday, Salim, Whittle, & Willett, 2003). When the Tanimoto Coefficient between the query and a compound is greater than or equal to the similarity threshold [0.9 (or 90%) by default], the compound is considered to be similar to the query and returned as a hit. The similarity threshold is adjustable. If the similarity threshold is increased [e.g., to 0.99 (or 99%)], the similarity search returns a smaller number of hit compounds that are more similar to the query. If a lower threshold is used [e.g., 0.85 (or 0.85%)], the search gives a greater number of hits that are more diverse, but less similar to the query.
4 Click the “Filters” button (‘4’ in Fig. 3) and refine the hits to only drug‐like compounds that satisfy Lipinski's rule of five.
  When the “Filters” button is clicked, the interactive histograms for some important molecular properties will be shown. Changing the minimum and maximum values of each property limits the hits to those compounds whose values for that property are within the specified range.
  Lipinski's rule of five (Lipinski et al., 1997) evaluates the drug‐likeness of a chemical, based on chemical and physical properties important for the pharmacokinetics of the chemical (e.g., its absorption, distribution, metabolism, and excretion in the human body). According to Lipinski's rule of five, an orally active drug typically has the following properties:
  - *A molecular weight less than 500 g/mol*
  - *No more than 5 hydrogen bond donors*
  - *No more than 10 hydrogen bond acceptors*
  - *An octanol‐water partition coefficient (log P) that does not exceed 5*.
    Although PubChem has experimental log P values for more than 26,000 compounds, this corresponds to a very small fraction of the 100+ million compounds in PubChem, and it is not practical to use the experimental log P values as a filter to refine the search results. Therefore, for this purpose, PubChem uses computed log P values, called “XLogP” (Cheng et al., 2007). The XLogP values are available for more than 90% of compounds in PubChem (except for inorganic and organometallic compounds).
5 Click the “Download” button (‘5’ in Fig. 3) to save the hit list as a CSV file for further analysis.
  The downloaded file contains the list of hit compounds and their computed molecular properties (such as molecular weight, heavy atom count, rotatable bond count, hydrogen bond donor and acceptor counts, polar surface area, molecular complexity, and XLogP). It also contains additional information [e.g., the assays in which the compounds were tested, the digital object identifiers (DOIs) for the articles that mention the compounds, the creation dates of the compound records, etc.]. This file can be loaded into a spreadsheet program (e.g., Microsoft Excel and Google Sheets) or a computer script (e.g., written in python or R) for further analysis.

## Basic Protocol 3 FINDING COMPOUNDS SIMILAR TO A QUERY COMPOUND THROUGH 3‐D SIMILARITY SEARCH

PubChem's search interface supports both 2‐D and 3‐D similarity searches. The molecular similarity methods used for the two similarity searches are complementary to each other. That is, one method can often recognize structural similarity that is unnoticed by the other approach. A brief overview of the underlying methods used in the 3‐D similarity search is given in Background Information.

In Basic Protocol 3, a 3‐D similarity search is performed to find the compounds structurally similar to losartan based on 3‐D similarity scores, and the 3‐D structures of the returned compounds are downloaded in a structure‐data file (SDF) format. The downloaded SDF file can be opened in popular 3‐D molecular viewers. Note that these 3‐D structures are not experimentally determined, but computationally generated as described in detail in previous papers (Bolton, Kim, & Bryant, 2011a; Kim, Bolton, & Bryant, 2013).

### Materials

An up‐to‐date Web browser, such as Google Chrome, Microsoft Edge, Safari, or Firefox, is required for this protocol (and all other protocols in this article)

**Figure 4:**
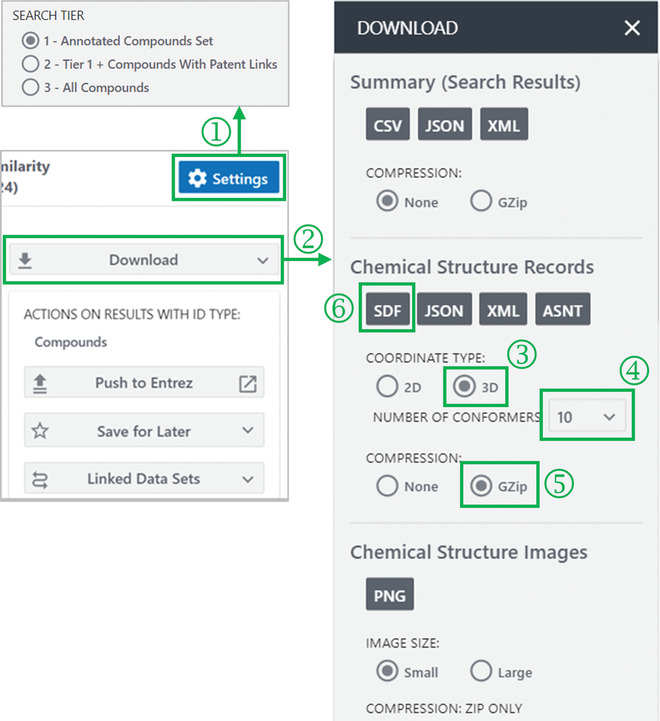
The Settings button available for the 3‐D similarity search and the download button for compound records. The Settings button (1) allows users to select the compound tiers against which the 3‐D similarity search is performed (see the main text for the three‐tiered 3‐D structure search). The download button (2) allows for downloading compound records in various file formats. To download up to 10 conformers per compound in a compressed structure‐data file (SDF) format, select “3D” for coordinate type (3), “10” for the number of conformers (4), and “gzip” for compression (5), and click the “SDF” button (6).

1 Repeat steps 1‐2 of Basic Protocol 2 to perform a structure search with losartan as a query.
2 Click the “3D Similarity” tab to view the hit list for the 3‐D similarity search (the purple box in Fig. 3).
  Because molecules can have multiple conformers, the 3‐D similarity score between two molecules is determined by selecting the highest score from the 3‐D similarity scores computed for all possible conformer pairs arising from the molecules. While up to ten conformers per compound are available for 3‐D similarity computation, it is not practical to perform a 3‐D similarity search against all compounds using up to ten conformers per compound, because a 3‐D similarity search is much slower and more resource‐intensive. Therefore, to find most information‐rich hits in a reasonable response time, a three‐tier approach is introduced for 3‐D similarity search. In this approach, compounds are classified into three tiers, based on their information content, and different numbers of conformers per compound are used during 3‐D similarity search:
  - *Tier 1: Compounds with annotations, using up to ten conformers per compound*
  - *Tier 2: Compounds with patent links, using up to five conformers per compound*
  - *Tier 3: All remaining compounds, using up to three conformers per compound*.
    By default, a 3‐D similarity search is performed against only the Tier 1 compounds (using up to ten conformers per compound). The search can be extended to the Tier 2 or Tier 3 compounds using the “SETTINGS” button (‘1’ in Fig. 4), but a smaller number of conformers per compound will be used.
    Also, note that it is not possible to adjust the 3‐D similarity search threshold, in contrast to the 2‐D similarity search threshold (see Basic Protocol 2). During the 3‐D similarity search, two compounds are considered to be similar if any conformer pair arising from them has a shape‐Tanimoto (ST) score of ≥0.80 (or 80%) and a color‐Tanimoto (CT) of ≥0.50 (or 50%) (Bolton, Kim, & Bryant, 2011b; Kim, Bolton, & Bryant, 2016). More information on the 3‐D similarity method used in PubChem can be found in Background Information.

3 Click the “Download” button (‘2’ in Fig. 4).
4 To save the 3‐D structures of hit compounds in an SDF format, select “3D” for the coordinate type, “10” for the number of conformers per compound, “gzip” for compression, and “SDF” for file format (‘3’ through ‘6’ in Fig. 4).
  PubChem generates a conformer model for each compound if it satisfies the following criteria:
  - *Not too large (with ≤50 non‐hydrogen atoms)*
  - *Not too flexible (with ≤15 rotatable bonds)*
  - *Has fewer than six undefined atom or bond stereocenters*
  - *Has only a single covalently bonded unit (i.e., not a salt or a mixture)*
  - *Consists of only supported organic elements (H, C, N, O, F, Si, P, S, Cl, Br, and I)*
  - *Contains only atom types recognized by the MMFF94s force field (Halgren, 1996*, *1996a**b*, *1999**)*.
    About 87% of compounds have computationally generated conformer models, and if a compound in the hit list does not have a conformer model, that compound will be ignored for download. While each of these conformer models contains up to 500 conformers, only up to 10 conformers per compound are accessible by the public. More detailed information on conformer generation in PubChem can be found in previous papers (Bolton et al., 2011a; Kim et al., 2013).

## COMPUTING SIMILARITY SCORES BETWEEN COMPOUNDS

Basic Protocols 2 and 3 demonstrate how to find compounds that are structurally similar to a query compound based on 2‐D and 3‐D similarity scores, respectively. However, the data returned from the similarity searches do not include the similarity scores between the query and the returned compounds. These scores can be used to sort the hit compounds and find higher‐ranked compounds within the list. They can also be used to perform a cluster analysis to identify important structural patterns of the hit compounds.

In this Support Protocol, we download the 3‐D similarity scores for the compounds returned from a 2‐D similarity search (in Basic Protocol 2) using the PubChem Score Matrix Service (https://pubchem.ncbi.nlm.nih.gov/score_matrix). The PubChem Score Matrix Service computes 2‐D and 3‐D similarity scores between compounds in PubChem. This service takes a list of M compounds and another list of N compounds as an input, computes similarity scores for M × N compound pairs arising from the combination of the two lists, and returns the scores in a matrix form or in a list of CID‐CID‐score triples. When only one list (of M compounds) is provided as an input, similarity scores are computed for M(M+1)/2 unique CID pairs, arising from the combination of the M compounds.

### Materials

**Figure 5:**
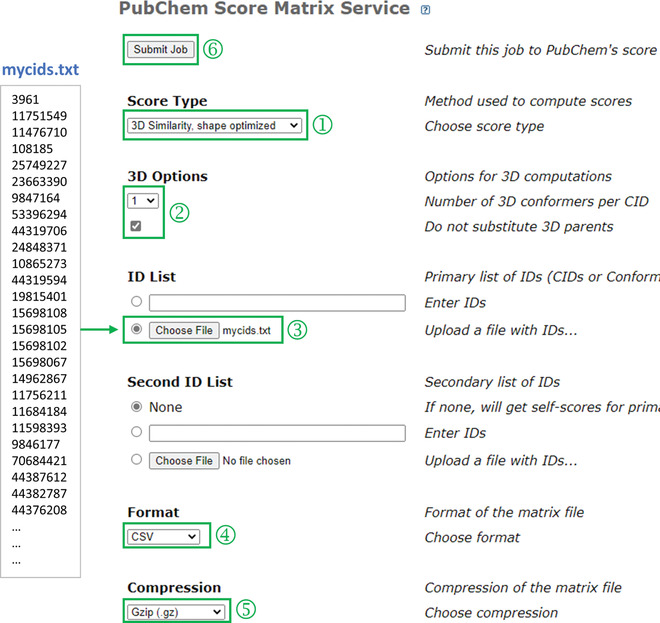
Computing similarity scores between compounds, using the PubChem Score Matrix Service (https://pubchem.ncbi.nlm.nih.gov/score_matrix/). One of three score types (2‐D similarity as well as shape‐ and feature‐optimized 3‐D similarities) can be selected through a dropdown menu (1). Additional options (2) are available for 3‐D similarity score computation. The list(s) of CIDs for similarity score computation can be provided in a text box or uploaded in a file (3). The output format (4) and compression method (5) can be selected through dropdown menus. Clicking the “Submit Job” button starts the similarity score computation.

An up‐to‐date Web browser, such as Google Chrome, Microsoft Edge, Safari, or Firefox, is required for this protocol (and all other protocols in this article)
In addition, this protocol requires a text file containing the CIDs of the hit compounds returned from Basic Protocol 2. This file can be generated from the CSV file downloaded in Basic Protocol 2. Open the CSV file in spreadsheet software (e.g., Microsoft Excel or Google Sheets). Copy the first column containing the CIDs (except for the column header), paste them into a text editor (e.g., Notepad on Windows PC and TextEdit on Mac), and save them as a text file. In this protocol, the file name is assumed to be mycids.txt. Double‐check that the file has the same format as the mycids.txt file in Figure 5 (e.g., one CID for each line).

1 Go to the PubChem Score Matrix Service (https://pubchem.ncbi.nlm.nih.gov/score_matrix).
  This page can also be reached via PubChemDocs (https://pubchemdocs.ncbi.nlm.nih.gov), which contains PubChem's help documents. It also serves as an entry point to various PubChem services. The help page for the Score Matrix Service can be found under the “Search and Analysis” section of PubChemDocs, and this page has a link to the Score Matrix Service.
2 Select “3D Similarity, shape optimized” for the score type (‘1’ in Fig. 5).
  Three similarity measures are supported: one 2‐D similarity measure and two 3‐D similarity measures (shape‐optimized and feature‐optimized). For more details about these similarity measures, see Background Information.
3 Select “1 conformer per CID” and check the “Do not substitute 3D parents” box (‘2’ in Fig. 5).
  Up to ten conformers per compound can be considered during the 3‐D similarity computation. Note that some compounds do not have conformer models, as mentioned in Basic Protocol 3. For example, PubChem does not generate a conformer model for salts and mixtures, but their parent forms may have 3‐D models. A parent compound is conceptually the "important" part of the molecule when the molecule has more than one covalently bonded unit. Specifically, a parent component must have at least one carbon and contain at least 70% of the heavy (non‐hydrogen) atoms of all the unique covalently bonded units (ignoring stoichiometry).
  By default, if a given CID does not have a 3‐D conformer model, but its parent structure does, the parent CID will automatically be substituted in the matrix. Checking the "do not substitute 3D parents" box disables this substitution and returns results for only the requested CIDs with 3‐D conformer models.
4 Select the text file containing the input CID list (i.e., mycids.txt; ‘3’ in Fig. 5).
  The input CID list can be uploaded in a file or typed into the text box. When only one list is provided, the similarity scores between compounds within the list are computed. If a second CID list is given, the similarity scores are computed for all CID‐CID pairs arising from the combination of the two CID lists.
  Note that there is a limit on the size of the score matrix that this service can handle. Currently, for 2‐D similarity computation, both the primary and optional compound lists should have no more than 10,000 compounds, and the number of compound pairs to consider should not exceed 1,000,000. For 3‐D similarity computation, the limits are no more than 10,000 “conformers” for both lists and no more than 1,000,000 “conformer pairs.. Therefore, if 3‐D similarity computation fails due to the size limit, it may be necessary to reduce the number of conformers to consider (‘2’ in Fig. 5).
5 Select “CSV” for format and “gzip” for compression (‘4’ and ‘5’ in Fig. 5) and click “Submit Job (‘6’ in Fig. 5).
  This step would take several minutes because 3‐D similarity computation is very time‐consuming.

## Basic Protocol 4 GETTING THE BIOACTIVITY DATA FOR THE HIT COMPOUNDS FROM SUBSTRUCTURE SEARCH

**Figure 6:**
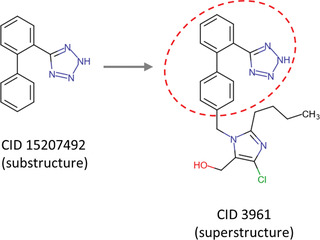
The concept of the substructure and superstructure. The structure of CID 15207492 (substructure) appears as a part of CID 3961 (superstructure).

When a chemical structure pattern appears in a bigger chemical structure, the former is called a substructure and the latter is referred to as a superstructure (see Fig. 6). In this protocol, a substructure search is performed to find compounds with a given substructure, and their bioactivity data are downloaded on a local computer. The downloaded data can be used in further analysis by means of third‐party software packages. This protocol uses two important features of PubChem's search interface, the PubChem Sketcher for structure input and the “Linked Data Sets” button for quick retrieval of linked data.

Previously, in Basic Protocols 2 and 3, a chemical name search (i.e., losartan as a query) was first performed to find the corresponding compound (CID 3961), which was used to specify the input chemical structure for a subsequent 2‐D and 3‐D similarity search. However, this approach cannot be used when the query structure does not exist in PubChem or when its name is unknown or ambiguous. In this case, the input structure can be provided by drawing it in the PubChem Sketcher.

This protocol also exemplifies the usefulness of linked data in PubChem. As mentioned in the Introduction, PubChem has multiple data collections. Some users often need to retrieve records in one data collection that are related to those in another data collection. For example, the present protocol retrieves bioactivity data (in BioAssay) associated with a list of chemicals (in Compound). This task can be done seamlessly with the “Linked Data Sets” button available on the search result page.

### Materials

An up‐to‐date Web browser, such as Google Chrome, Microsoft Edge, Safari, or Firefox, is required for this protocol (and all other protocols in this article)

**Figure 7:**
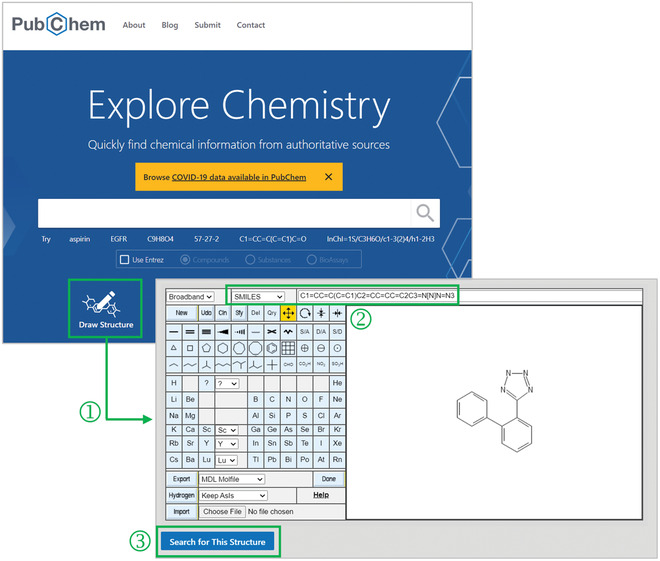
Using the PubChem Sketcher to provide a query structure for chemical structure searches. The PubChem sketcher can be accessed from the PubChem homepage through the “Draw Structure” button (1). The query structure can be drawn manually or converted from a line notation like a SMILES or InChI string (2). Clicking the “Search for This Structure” button (3) initiates the structure searches.

1 Go to the PubChem homepage (https://pubchem.ncbi.nlm.nih.gov) and launch the PubChem Sketcher by clicking the “Draw Structure” button (‘1’ in Fig. 7).

**Figure 8:**
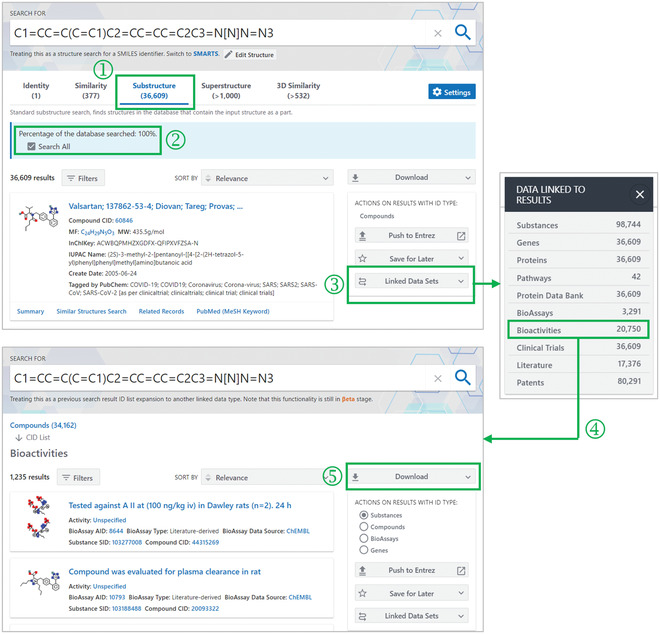
Retrieving bioactivity data for the compounds returned from substructure search. When the input structure is provided, PubChem performs multiple types of structure search. The results of the substructure search can be viewed by clicking the “Substructure” tab (1). By default, structure search stops when it finds 1000 hit compounds. If the user wants to find more than 1000 hit compounds, it is necessary to check the “Search All” box (2). The bioactivity data for the hit compounds can be retrieved by clicking the “Linked Data Sets” button available on the right column (3) and then selecting “Bioactivities” from the popup menu (4). The bioactivity data can be downloaded through the “Download” button (5).

2 Draw the structure of 5‐(2‐phenylphenyl)‐2H‐tetrazole, by providing its SMILES string C1=CC=C(C=C1)C2=CC=CC=C2C3=N[N]N=N3 in the text box available at the top of the Sketcher (‘2’ in Fig. 7).
  While the user can draw the input structure manually, it is possible to generate the input structure from a line notation like a SMILES or InChI string. SMILES arbitrary target specification (SMARTS) strings (Daylight Chemical Information Systems Inc.; see Internet Resources) and InChIKeys (Heller et al., 2015) can also be used. This functionality is very useful especially when the input structure is too big or complex to draw manually.
3 After drawing the input structure, click the “Search for This Structure” button (‘3’ in Fig. 7).
  The query will be used for multiple types of structure searches and, by default, the result from the identity search is displayed. The user can move to the results for other types of searches by clicking the appropriate tabs.
4 Click the “Substructure” tab to view the hit compounds from the substructure search (‘1’ in Fig. 8).

5 Check the “Search All” box (‘2’ in Fig. 8) to extend the search to all compounds in PubChem.
  In general, a structure search is very time‐consuming and resource‐intensive. Therefore, by default, each type of structure search stops when a maximum of 1000 hit compounds are found, and the search result is displayed with a message that indicates what percentage of the database is searched. Clicking the “Search All” box below this message extends the search to the remaining part of the database.
  When a query for substructure search is too small or too generic, it will result in too many hit compounds for PubChem to handle. Therefore, the maximum number of hits that can be returned from a structure search is limited to 1,000,000.
6 Click the “Linked Data Sets” button on the right column (‘3’ in Fig. 8) and select the “Bioactivities” link from the pop‐up menu (‘4’ in Fig. 8).
  Through the Linked Data Sets button, the user can access various types of data associated with the hit records. In this example, the bioactivity data for the hit compounds returned from the substructure search are retrieved.
7 Click the Download button to save the linked data on a local computer (‘5’ in Fig. 8).
  The downloaded data contains the AIDs, activity outcomes, activity concentrations, activity names, and other related information. The downloaded bioactivity data, in conjunction with molecular structure information, can be used for developing a structure‐activity relationship model.

## Basic Protocol 5 FINDING DRUGS THAT TARGET A PARTICULAR GENE

While it is possible to retrieve all macromolecules interacting with a given chemical (as done in Basic Protocol 1), the user may want to find all chemicals that interact with a given gene or protein. This task can be done through the Summary page of a gene or protein record, which presents all PubChem data related to that macromolecule. It includes not only known drugs and tested chemicals, but also annotations collected from major gene or protein information resources.

Basic Protocol 5 aims to find all known drugs that interact with the gene encoding the human type‐1 angiotensin II receptor, which is the target of losartan (see Basic Protocol 1). This protocol begins with a text search using the gene name as a query. Then, the resulting gene list is filtered based on taxons to identify the gene for humans. The Summary page of this gene contains lists of drugs targeting it, which are collected from DrugBank (Wishart et al., 2018), ChEMBL (Mendez et al., 2019), and IUPHAR/BPS Guide to PHARMACOLOGY (Armstrong et al., 2020). These lists can be downloaded on a local computer.

### Materials

An up‐to‐date Web browser, such as Google Chrome, Microsoft Edge, Safari, or Firefox, is required for this protocol (and all other protocols in this article)

**Figure 9:**
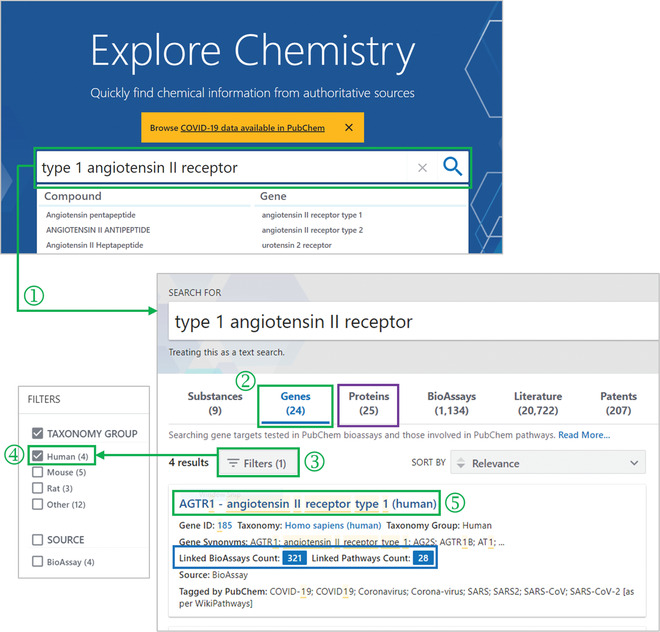
Search by gene/protein name using “type 1 angiotensin II receptor” as an example. When a gene/protein name is used as a query (1), multiple collections are searched. Clicking the “Genes” tab shows the gene records returned from the search (2). To view hit protein records, click the “Proteins” tab (indicated in the purple box). The filter (3) allows for selecting only the human gene records (4). Clicking the human *AGTR1* gene (5) directs the user to its Summary page. Note that gene records may have associated bioassay and/or pathway records in PubChem (as indicated in the blue box).

1 Go to the PubChem homepage and perform a text search with type 1 angiotensin II receptor as a query (‘1’ in Fig. 9).
  When a text query consists of multiple words separated by blanks, the query is interpreted in such a way that a Boolean AND operator is applied between the words. That is, the query vitamin C is interpreted as vitamin AND C, and retrieves records that contain the strings vitamin and C together. To search for the phrase vitamin C, the query should be enclosed in double quotes. With that said, the following queries will be interpreted differently:
  - *type 1 angiotensin II receptor*
  - *type‐1 angiotensin II receptor*
  - *“type 1 angiotensin II receptor” (enclosed in double quotes)*
  - *“type‐1 angiotensin II receptor” (enclosed in double quotes)*
  - *“angiotensin II receptor type 1” (enclosed in double quotes)*
  - *“angiotensin II receptor type‐1” (enclosed in double quotes)*.
    Among these examples, the first one is used as a query in Basic Protocol 5, as shown in Figure 9. It is interpreted as “type AND 1 AND angiotensin AND II AND receptor” and returns any records containing the five words. If the query needs to be interpreted as a phrase (e.g., “type 1 angiotensin II receptor”) to identify more specific hits, the query should be enclosed in double quotes. In this case, however, the search would miss records containing a phrase like “angiotensin II receptor type 1”.

**Figure 10:**
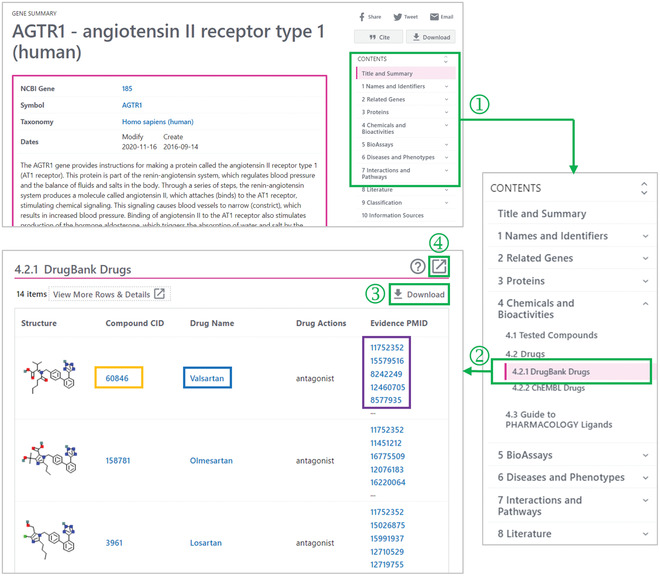
Using the Gene Summary page for the human type‐1 angiotensin II receptor (https://pubchem.ncbi.nlm.nih.gov/gene/185) to find drugs targeting the gene (or the proteins that it encodes). The Table of Contents on the right column (1) can be used to navigate the Gene Summary page. Clicking the “DrugBank Drugs” (2) directs the user to the section that contains information on drugs targeting the gene, curated by DrugBank. The information presented in this section can be downloaded (3). The Full‐screen view button (4) presents additional information in a full‐screen view mode. For each drug, there are links to the corresponding records in the PubChem Compound and DrugBank (indicated in the yellow and blue boxes, respectively) as well as links to the PubMed records that provide the evidence of the drug‐target information (indicated in the purple box).

2 Select the “Genes” tab (‘2’ in Fig. 9) to display the search result from the gene collection.
  As mentioned in Basic Protocol 1, when a text query is provided, multiple data collections are searched simultaneously. Note that the query “type 1 angiotensin II receptor” may be viewed as a protein name or the gene encoding it. Because the objective of Basic Protocol 5 is to download the drugs interacting with the gene, the “Gene” tab is clicked.
3 Click the Filters button (‘3’ in Fig. 9) and select “Human” under the taxonomy group (‘4’ in Fig. 9).
  Searching PubChem often results in a large number of hits. The search results can be narrowed down by filtering them based on certain attributes, as shown in Basic Protocol 2, where the hit compounds are filtered based on several molecular properties, such as molecular weight, hydrogen bond donor and acceptor counts, XLogP, etc. For the Gene collection, hit records can be filtered by taxonomy group (e.g., human, mouse, rat, and other) and data source type (e.g., bioassay and pathway). Note that the data source type filter allows the user to filter the genes based on whether they have associated bioassays or pathway records. The Gene collection contains: (1) those genes that have been tested against in any bioassay archived in PubChem, and/or (2) those that are involved in a pathway archived in PubChem. For example, as indicated in the blue box in Figure 9, the human angiotensin II receptor type 1 has been tested in more than 300 bioassays and is associated with more than 20 pathway records.
4 Click the gene record for the human angiotensin II receptor type 1 (‘5’ in Fig. 9).
  Clicking this gene record directs the user to its Gene Summary page. The Gene Summary page contains a wide variety of information on the gene. This includes the gene names, symbols, identifiers, and classifications, as well as the structure and function of the proteins encoded by the gene. The Gene Summary page also contains information on related chemicals, drugs, bioassays, pathways, and diseases, along with links to relevant scientific articles. This page has cross‐links to related records in other PubChem data collections as well as resources external to PubChem.
5 Use the Table of Contents (‘1’ in Fig. 10) in the right column to go to the DrugBank Drugs subsection (‘2’ in Fig. 10).
  This subsection presents a list of the drugs associated with the human type 1 angiotensin II receptor, along with their CIDs, names, and the PMIDs for relevant articles. Clicking CIDs, names, and PMIDs in this table (the yellow, blue, and purple boxes in Fig. 10) directs the user to the page for the corresponding record in PubChem, DrugBank, and PubMed, respectively.

6 Click the Download button (‘3’ in Fig. 10) to download the data.
  The downloaded CSV file contains additional information about the drugs.
7 If necessary, click the “Full‐view” button (‘4’ in Fig. 10) to get more detailed information.
8 Get the drug information from ChEMBL in a similar way to that described in steps 5 through 7. This information can be found in the “ChEMBL Drugs” section.
9 Get the drug information from Guide To PHARMACOLOGY in a similar way as described in steps 5 through 7. This information can be found in the “Guide to PHARMACOLOGY Ligands” section.
  The drug lists from the three sources (DrugBank, ChEMBL, and Guide to PHARMACOLOGY) are not the same, while some drugs appear in all three lists. In general, each data source has its own focus area, drug/chemical coverage, data content, and curation strategy. Therefore, cross‐checking data from multiple sources is a good practice.

## Basic Protocol 6 GETTING BIOACTIVITY DATA OF ALL CHEMICALS TESTED AGAINST A PROTEIN

Basic Protocol 6 is designed to demonstrate how to download the bioactivity data of all chemicals tested against a given protein and how to quickly access data for a protein orthologous to another protein, using the human type‐1 angiotensin II receptor and its rat ortholog as an example. This protocol is similar to Basic Protocol 5, which downloads the list of drugs interacting with the gene encoding type‐1 angiotensin II receptor. However, it should be kept in mind that a gene record in PubChem can be associated with multiple protein records, reflecting the fact that a gene can produce multiple protein sequences (e.g., isoforms or variants). Because bioassays archived in PubChem were performed typically against one of the multiple protein sequences that may arise from a single gene, the Summary pages of the different proteins from the same gene present different sets of bioactivity data. These data are merged together and presented on the Summary page of the encoding gene. Therefore, extra care should be taken when downloading the bioactivity data from the Summary page of a gene or protein.

### Materials

An up‐to‐date Web browser, such as Google Chrome, Microsoft Edge, Safari, or Firefox, is required for this protocol (and all other protocols in this article)

**Figure 11:**
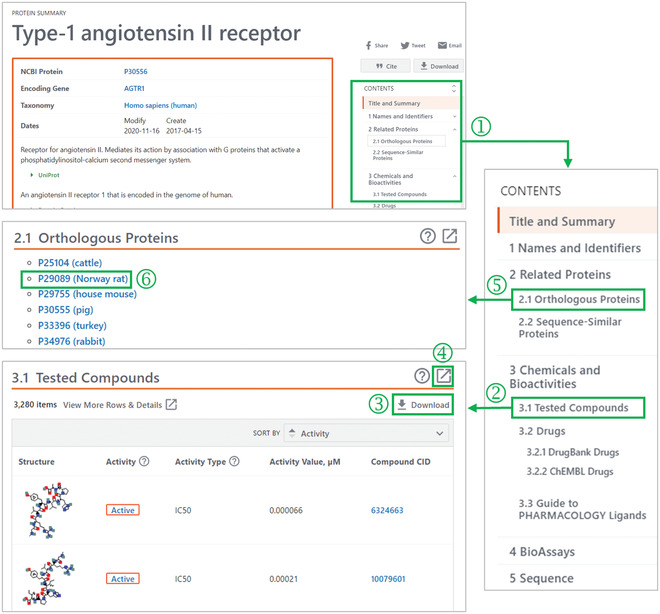
Using the Protein Summary page for the human type‐1 angiotensin II receptor (https://pubchem.ncbi.nlm.nih.gov/protein/P30556) to find compounds tested against the protein and its rat orthologs. This page can be navigated using the Table of Contents on the right column (1). Clicking the “Tested Compounds” (2) directs the user to the “tested compound” section. The bioactivity data for these compounds against the target protein can be downloaded through the “Download” button (3), and additional information can be viewed by clicking the “Full‐screen view” button (4). A list of the orthologs of the protein can be accessed by clicking the “Orthologous Proteins” section (5). Clicking “P29089 (Norway rat)” in this section (6) leads to its Protein Summary page, where information on tested compounds against the rat orthologs can be found.

1 Go to the Protein Summary page of the human type‐1 angiotensin II receptor. This can be done in a similar manner to steps 1 through 4 of Basic Protocol 5 (Fig. 9), except that the “Proteins” tab (the purple box in Fig. 9) should be clicked to access the hit protein records instead of the gene records.
2 Use the Table of Contents (‘1’ in Fig. 11) on the right column to go to the Tested Compounds subsection (‘2’ in Fig. 11).
  This data table contains the tested compounds, activity outcomes (e.g., active, inactive, inconclusive, or unspecified), and activity types and values (e.g., IC_50_, EC_50_, K_i_, K_d_, etc.). Usually, these data are not from a single assay, but from multiple assays. It means that a compound may occur multiple times in this table, because it can be tested in multiple assays. These assays were likely to be performed under different experimental conditions and using different experimental methods. Also, the criteria used to determine whether a compound is active or not are different among the assays. Therefore, care should be taken when interpreting these data.

3 Download the list of the tested compounds with their bioactivity data against the target protein (‘3’ in Fig. 11).
  The downloaded CSV file contains more detailed information presented in the data table of the Protein Summary page. For bioactivity data derived from a scientific article, the corresponding PMID is also included in the downloaded file.
4 If necessary, click the “Full‐view button” (‘4’ in Fig. 11) to get more detailed information.
5 Go to the Orthologous Proteins section (‘5’ in Fig. 11) and click “P29089 (Norway rat)” (‘6’ in Fig. 11). This leads the user to the Summary page for the orthologous protein in rats.
6 Repeat steps 2 through 4 to download the list of the tested compounds and their bioactivity data for the rat type‐1 angiotensin II receptor.

## Basic Protocol 7 FINDING COMPOUNDS ANNOTATED WITH CLASSIFICATIONS OR ONTOLOGICAL TERMS

PubChem records are annotated with various classifications and ontological terms. For example, losartan (CID 3961) is annotated with three Medical Subject Headings (MeSH) terms, “Angiotensin II Type 1 Receptor Blockers”, “Antihypertensive Agents”, and “Anti‐Arrhythmia Agents”, as shown at https://pubchem.ncbi.nlm.nih.gov/compound/3961#section=MeSH‐Pharmacological‐Classification.

PubChem users often want to access all records annotated with a particular term. This task can be done using the PubChem Classification Browser, which can be accessed from the PubChem homepage or via https://pubchem.ncbi.nlm.nih.gov/classification/.

The classification browser allows users to browse the distribution of PubChem records among nodes in the hierarchy of ontological terms and classifications and subset PubChem records annotated with the desired term.

In this protocol, the Classification Browser is used to retrieve chemicals with the same therapeutic uses as losartan, based on the MeSH annotations (that is, chemicals that are known as both antihypertensive and antiarrhythmic agents). This involves performing two independent searches (one for antihypertensive agents and the other for antiarrhythmic agents) and finding chemicals returned in both searches. PubChem users often need to perform a series of searches, followed by taking the intersection or union of the search results or identifying records returned from one search, but not from another. These tasks can be done in PubChem using Boolean operators (AND, OR, and NOT), as exemplified in this protocol.

### Materials

An up‐to‐date Web browser, such as Google Chrome, Microsoft Edge, Safari, or Firefox, is required for this protocol (and all other protocols in this article)

**Figure 12:**
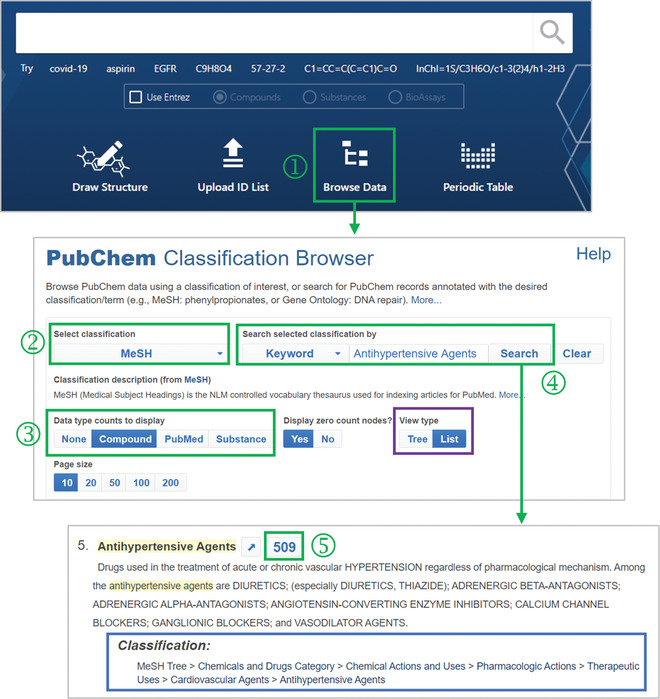
Finding records annotated with classification and ontological terms, using the PubChem Classification Browser (https://pubchem.ncbi.nlm.nih.gov/classification/). The classification browser can also be accessed by clicking the “Browse Data” button (1), available on the PubChem homepage. To find compounds annotated with the Medical Subject Headings (MeSH) terms “Antihypertensive Agents”, select “MeSH” for classification (2), “Compound” for data type counts to display (3), and type “Antihypertensive Agent” in the search box (4). Clicking the compound record count (5) for the MeSH term will show the relevant records (see Fig. 13). Note that MeSH terms are organized in a hierarchical (tree) structure (as indicated in the blue box). The view type menu (indicated in the purple box) allows the user to select to view the returned MeSH terms in a list or tree view.

1 Go to the PubChem homepage and click the “Browse Data” icon below the search box (‘1’ in Fig. 12). This leads to the Classification Browser, which can also be accessed directly via https://pubchem.ncbi.nlm.nih.gov/classification/.

**Figure 13:**
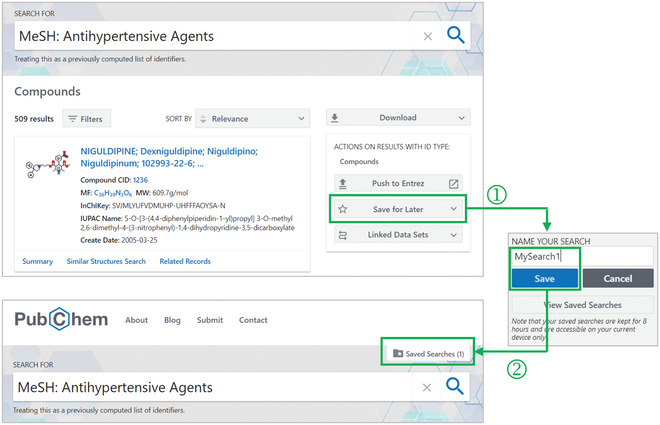
Saving a search result for later use. A search can be saved by clicking the “Save for Later” button (1) and giving an alias to it (2). When it is saved successfully, the “Saved Search” button appears above the search box.

2 Select “MeSH” from the “Select classification” dropdown menu (‘2’ in Fig. 12).
  The Classification Browser supports various classifications and ontologies, including, but not limited to:
  - *Medical Subject Headings (see Internet Resources)*
  - *ChEBI Ontology (Hastings et al.*, *2016**)*
  - *Gene Ontology (Ashburner et al.*, *2000*; *Carbon et al.*, *2021**)*
  - *Food and Drug Administration (FDA) Pharmacological Class (FDA*, *2021**)*
  - *WIPO (World Intellectual Property Organization) International Patent Classification (WIPO*, *2021**)*
  - *World Health Organization (WHO) Anatomical Therapeutic Chemical (ATC) classification system (WHO*, *2021**)*
  - *PubChem Compound Table of Contents (TOC)*.
    The PubChem Compound TOC is also available in the Classification Browser. This allows users to quickly identify and retrieve compounds that have a particular kind of annotation (e.g., those with solubility data, those with toxicological information, those which have been tested in a clinical trial, those mentioned in scientific articles or patent documents, etc.).
3 Select the “Compound” from the “Data type counts to display” menu (‘3’ in Fig. 12).
  This dropdown menu allows users to select the desired type of record. In this example, the “Compound” option is selected because we want to find “compounds” annotated with the MeSH term “Antihypertensive Agent”. If we want to find “articles” about antihypertensive agents, the “PubMed” option should be selected. Note that the available options under this menu vary depending on the classification selected (‘2’ in Fig. 11). For example, if the WIPO's International Patent Classification is selected for the classification, the “Patent” option will appear for the data type menu.
4 Type Antihypertensive Agents in the search box (‘4’ in Fig. 12).
  This search box has an autocomplete/autosuggestion function to assist users in providing the input keyword. This box can accept either a keyword or an identifier as an input. To provide an identifier, an appropriate type of identifier should be selected from the dropdown menu next to the text search box.
5 From the returned hit list, find the “Antihypertensive Agents” node and click the record count for that node (‘5’ in Fig. 12).
  As implied in the blue box in Figure 12, each returned record corresponds to a node in a classification tree. The returned hits can be presented in two different ways (the Tree view and List view), and the user can move between the two views by selecting either “Tree” or “List” from the “View type” menu (indicated by the purple box in Fig. 12).
6 The previous step leads to a web page that shows compounds annotated as antihypertensives (Fig. 13). Save this list by clicking the “Save for Later” button available on the right column and providing an alias for that list (e.g., “MySearch1”) (‘1’ in Fig. 13). When the list is successfully saved, a new button “Saved Searched (1)” appears above the search box (‘2’ in Fig. 13).
  The message presented in the Search Box of the bottom panel in Figure 13 is not the query that users can use.

**Figure 14:**
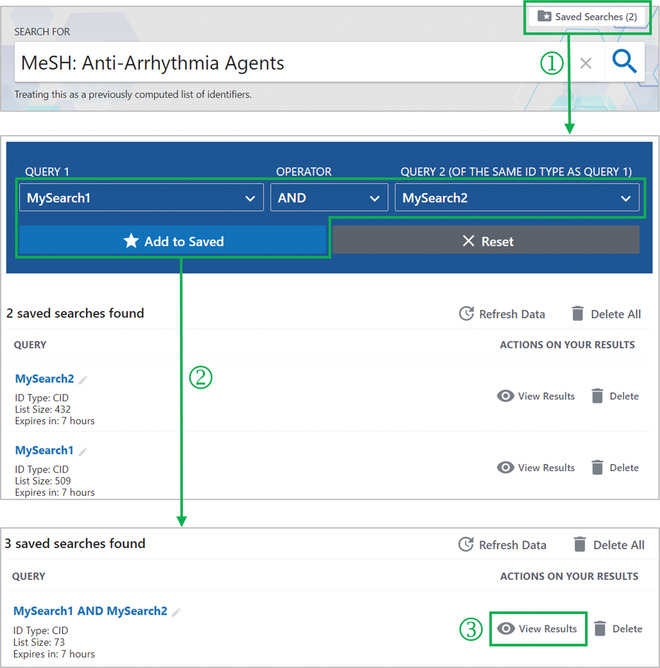
Combining saved searches to perform a complex search. Clicking the “Saved Searches” button (1) presents a dialog box in which saved searches can be combined using Boolean operators (AND, OR, and NOT). In this screenshot, two saved searches “MySearch1” and “MySearch2” are combined with the AND operator (2) and added to the list of saved searches. The resulting hits can be viewed by clicking the “View Results” button (3).

7 Repeat steps 1 through 6 to retrieve the list of compounds annotated with the MeSH term “Anti‐arrhythmia Agents” and save them as “MySearch2”.
  If both lists are saved correctly, a button “Saved Searches (2)” will appear above the search box as shown in Figure 14.

8 Click the “Saved Search (2)” button (‘1’ in Fig. 14). This launches a dialog box that enables users to perform advanced searches by combining results from previous searches using Boolean operators (AND, OR, and NOT).
  The saved results expire after 7 hr of inactivity.
9 Select the saved results, “MySearch1” and “MySearch2”, from the Query 1 and Query 2 dropdown menus and select “AND” from the Operator menu. Then, click the “Add to Saved” button (‘2’ in Fig. 14).
10 Click the “View Results” button to go to the web page that shows the resulting compound list (‘3’ in Fig. 14).

## Basic Protocol 8 GETTING STEREOISOMERS AND ISOTOPOMERS OF A COMPOUND THROUGH IDENTITY SEARCH

This protocol demonstrates how to find stereoisomers and isotopomers of a given compound, with valsartan (CID 60846) as an example. This task can be done using identity search, which is one of the structure search types supported by PubChem. An identity search returns compounds identical to the query molecule. While it may sound straightforward, the search results can vary, depending on what is meant by “identical” compounds. PubChem's identity search allows for some flexibility in the definition of chemical identity. By default, two molecules are considered identical if they have the same connectivity, isotopism, and stereochemistry [i.e., (R/S)‐configuration and cis/trans‐isomerism]. The user can change this behavior by choosing to ignore isotopism and/or stereochemistry. When stereochemistry is ignored, compounds with the same connectivity and isotopism, but with varying stereochemistry (i.e., stereoisomers), are returned. If isotopism is ignored, the identity search finds compounds with the same connectivity and stereochemistry, but with different isotopes (i.e., isotopomers). In this protocol, identity search is performed with different definitions of chemical identity to find stereoisomers and isotopomers of valsartan (CID 60846), which is a structural analog of losartan.

### Materials

An up‐to‐date Web browser, such as Google Chrome, Microsoft Edge, Safari, or Firefox, is required for this protocol (and all other protocols in this article)

**Figure 15:**
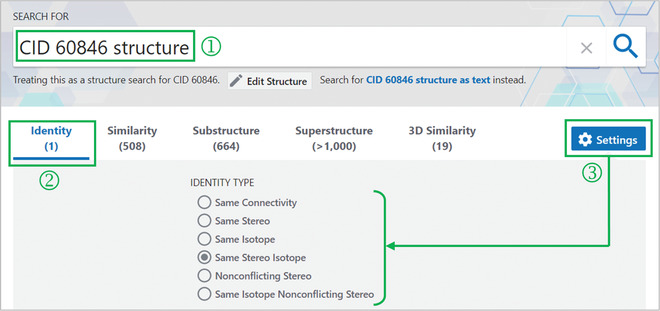
Performing an identity search. The query “CID 60846 structure” (1) initiates various types of structure searches using the structure of 60846 as a query. The result of the identity search can be viewed by the “Identity” tab (2). The Settings button allows users to select one of the several definitions of chemical identity (3).

1 Go to the PubChem homepage, type CID 60846 structure (‘1’ in Fig. 15), and hit the search button.
  The query CID 60846 structure invokes a chemical structure search for CID 60846. If CID 60846 is used alone as a query (without structure), it will direct the user to the Compound Summary page of CID 60846.

**Figure 16:**
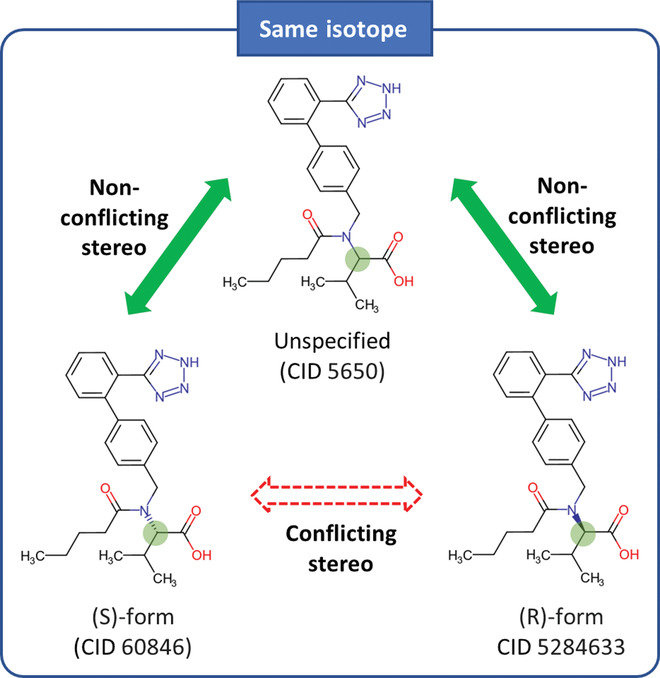
Compounds with conflicting and nonconflicting stereocenters.

2 Click the “Identity” tab (‘2’ in Fig. 15) and the “Settings” button (‘3’ in Fig. 15).
  This will show the options that control the definition of chemical identity. By default, the “Same Stereo Isotope” option is selected, meaning that the search returns compounds with the same connectivity, stereochemistry, and isotope. The PubChem chemical structure standardization process (Hähnke et al., 2018) ensures that chemical structures with the same connectivity, stereochemistry, and isotopism are assigned to an identical CID. Therefore, an identity search with the default option returns only one hit, the query itself, if the query molecule exists in the PubChem Compound database, or no hit if the query does not exist in Compound.
3 Select the “Same Isotope” option to find stereoisomers of valsartan.
  As mentioned previously, connectivity, isotopism, and stereochemistry are the three factors considered during an identity search. The “Same Isotope” option requires that identical compounds have the same connectivity and isotopes, but ignores stereochemistry. As a result, this option returns stereoisomers of the query molecule. For example, the query molecule (valsartan: CID 60846) has a chiral center in (S)‐configuration, and the “Same Isotope” option returns three compounds: the query itself [(S)‐form], CID 5284633 [(R)‐form], and CID 5650 (with the “unspecified” configuration at its chiral center) (see Fig. 16).
  Also, the identity search has options called “Nonconflicting Stereo” and “Same Isotope Nonconflicting Stereo” (Fig. 15). These options help the user to deal with the ambiguity arising from stereocenters with unspecified configuration. For example, the unspecified configuration at the chiral center of CID 5650 means that the compound may be an (R)‐form, (S)‐form, or both (e.g., racemic mixture). CID 5650 may or may not have the same stereochemistry as the (S)‐form (CID 60846), the query compound used for an identity search in this protocol. Therefore, these two CIDs are considered to have “nonconflicting” stereochemistry. In contrast, the (R)‐ and (S)‐forms have “conflicting” stereochemistry, because they cannot have the same stereochemistry. The “Nonconflicting Stereo” and “Same Isotope Nonconflicting Stereo” options allow the user to take into account this ambiguity concerning unspecified stereo configuration. If the “Same Isotope Nonconflicting Stereo” option (rather than “Same Isotope”) is used in this step, the identity search will return only two compounds, the query compound ((S)‐form) and CID 5650 (with unspecified stereochemistry).

4 Download the returned stereoisomers in a CSV format.
5 Select the “Same Stereo” option to find stereoisomers of valsartan.
  With this option, chemical identity will be assessed based on connectivity and stereochemistry, but isotopism will be ignored. As a result, this step results in the query and its various isotopomers.
6 Download the returned isotopomers in a CSV format.

## COMMENTARY

### Background Information

#### PubChem as an archive and a knowledgebase

PubChem (https://pubchem.ncbi.nlm.nih.gov; Kim, 2016; Kim et al., 2019; Kim et al., 2021; Kim et al., 2016) is a popular chemical information resource that plays a dual role as a data repository (archive) and a knowledgebase. As a data repository, PubChem needs to archive various types of chemical information provided by individual data contributors. As a knowledgebase, it should provide the user with easy access to comprehensive chemical data from authoritative sources. These two demands are taken into account in data organization in PubChem. As mentioned previously, PubChem has multiple data collections, including Substance, Compound, BioAssay, Gene, Protein, Pathway, and Patent. Among them, Substance and BioAssay play a role as an archive. Substance stores chemical information provided by individual data sources, and BioAssay archives the description and test results of biological assay experiments. Compound is a knowledgebase that provides comprehensive information on unique chemical structures extracted from Substance. The other data collections (i.e., Gene, Protein, Pathway, and Patent) are also knowledgebases that provide information on chemicals associated with a specific gene, protein, pathway, and patent document, respectively.

#### Chemical structure search in PubChem

Beyond chemical name searches (Basic Protocols 1), PubChem allows the user to search by chemical structure. The input chemical structure can be provided using line notations like SMILES (Weininger, 1988, 1990; Weininger et al., 1989) and InChI (Heller et al., 2015), or drawn using the PubChem Sketcher (Ihlenfeldt, Bolton, & Bryant, 2009). If the input structure exists in the PubChem Compound database, its CID can also be used as a query. Alternatively, the structure of a hit compound from a previous search can be also be used, as demonstrated in Basic Protocols 2 and 3). Various types of structure searches are supported, including identity search, 2‐D and 3‐D similarity searches, and substructure/superstructure searches.

##### Identity search

Through identity search (Basic Protocol 8), the user can find compounds identical to a query compound. While it seems straightforward, the identity search can result in different hits, depending on the definition of “identical compounds.” For example, while isotopically labeled glucose (with ^13^C and ^15^N atoms) have the same chemical and biological properties as non‐labeled one, they show different signals in nuclear magnetic resonance (NMR) or mass spectrometry (MS) experiments. Therefore, depending on the context, the two molecules may or may not be considered identical. PubChem's identity search allows the user to select one of several different contexts of “identity,” as demonstrated in Basic Protocol 8. By default, identity search returns compounds with the same connectivity, stereochemistry, and isotopism as the query molecule.

##### 2‐D and 3‐D similarity search

Similarity search returns compounds structurally similar to a query molecule (Basic Protocols 2 and 3). Because molecular similarity is a subjective concept, which is not physically measurable, various similarity methods have been proposed to quantify it. The most widely used ones are 2‐D similarity methods. In these approaches, the similarity between two molecules is evaluated by comparing their molecular fingerprints (binary fragment vectors encoding the 2‐D structures of molecules) and computing a similarity score, which quantifies how similar the molecules are. This score can be computed using various metrics, but the Tanimoto coefficient is the most popular choice. In another group of methods, called 3‐D similarity methods, 3‐D structures of molecules are superimposed to find the “best” overlap between them. While 3‐D similarity methods are much slower than 2‐D similarity methods, they often recognize molecular similarity that is not readily detected by 2‐D similarity methods. PubChem supports both 2‐D and 3‐D similarity searches. They usually give different lists of hit compounds, complementing each other. More detailed information on the 2‐D and 3‐D similarity methods used in PubChem is provided below.

##### Substructure and superstructure search

When a chemical structure occurs as a part of a bigger chemical structure, the former is called a substructure and the latter is referred to as a superstructure. For example, as shown in Figure 6, the structure of CID 15207492 (5‐(2‐phenylphenyl)‐*2H*‐tetrazole) occurs as a part of CID 3961. Therefore, CID 15207492 is a substructure of CID 3961.

In a substructure search, a substructure is provided as a query to find molecules that contain the substructure (that is, superstructures that contain the query substructure). On the contrary, superstructure search returns molecules that comprise or make up the provided superstructure query (that is, substructures that are contained in the query superstructure). PubChem supports both substructure and superstructure searches. It also provides flexible matching options that allow the user to specify how to deal with stereochemistry, isotopism, tautomerism, formal charges, aromatic bonds, and explicit hydrogens during the searches. Basic Protocol 4 demonstrates how to perform a substructure search using CID 15207942 as a query substructure.

#### 2‐D and 3‐D molecular similarity assessment in PubChem

This section provides a brief overview of the 2‐D and 3‐D similarity methods used in PubChem and more detailed information on them is given elsewhere (Bolton et al., 2011; Kim et al., 2016; Kim, Bolton, & Bryant, 2011). PubChem evaluates 2‐D molecular similarity using the PubChem substructure fingerprints (PubChem, 2009). They are 881‐bit‐long binary vectors, each bit of which represents the absence (0) or presence (1) of a particular structural characteristic found in a chemical structure, such as an element count, a type of ring system, atom pairing, and fragment patterns. The PubChem fingerprints are used to quantify 2‐D similarity between two compounds, in conjunction with the Tanimoto coefficient, as shown in Equation 1 (Chen & Reynolds, 2002; Holliday et al., 2002; Holliday et al., 2003):

(1) $$Tanimoto=\frac{N_{AB}}{N_{A}+N_{B}-N_{AB}}$$

where *N_A_* and *N_B_* are the counts of bits set in the fingerprints representing molecules A and B, respectively, and *N_AB_* is the count of common bits set in both fingerprints. While a Tanimoto coefficient ranges from 0 (for no similarity between molecules) to 1 (for identical molecules, relative to the resolution of the substructure fingerprint).

On the other hand, 3‐D similarity in PubChem is assessed using the Gaussian‐shape overlay method of Grant and Pickup (Grant & Pickup, 1995, 1996, 1997; Grant, Gallardo, & Pickup, 1996), implemented in the Rapid Overlay of Chemical Structures (ROCS; Rush, Grant, Mosyak, & Nicholls, 2005). This method quantifies two aspects of 3‐D similarity (i.e., shape similarity and feature similarity) between two conformers. The shape similarity is computed using the shape‐Tanimoto (ST) (OpenEye Scientific Software, 2010a, 2010b), as shown in Equation 2:

(2) $$ST=\frac{V_{AB}}{V_{AA}+V_{BB}-V_{AB}}$$

where *V_AA_* and *V_BB_* are the self‐overlap volumes of conformers A and B, respectively, and *V*_AB_ is the overlap volume between conformers A and B. The feature similarity is evaluated using the color‐Tanimoto (CT) (OpenEye Scientific Software, 2010a, 2010b), as shown in Equation 3:

(3) $$CT=\frac{\sum_{f}V_{AB}^{f}}{\sum_{f}V_{AA}^{f}+\sum_{f}V_{BB}^{f}-\sum_{f}V_{AB}^{f}}$$

where the index “*f*” indicates any of six “fictitious” feature (color) atom types (hydrogen bond donors and acceptors, cations, anions, hydrophobes, and rings.), $V_{AA}^{f}$ and $V_{BB}^{f}$ are the self‐overlap volumes of conformers A and B for feature atom type *f*, respectively, and $V_{AB}^{f}$ is the overlap volume between conformers A and B for feature atom type *f*. To consider the (steric) shape similarity and (chemical) feature similarity simultaneously, the combo‐Tanimoto (ComboT) is used, as indicated in Equation 4:

(4) ComboT=ST+CT

Because both ST and CT scores range from 0 (for no similarity) to 1 (for identical molecules), by definition, the ComboT score can have a value from 0 to 2 (without normalization).

To find the best superposition between molecules, two approaches can be used: shape optimization and feature optimization. The shape‐optimization approach finds the molecular superposition that maximizes the ST score and then computes the CT and ComboT scores at that superposition. On the other hand, the feature optimization approach considers the shape and feature simultaneously to find the best superposition.

It is noteworthy that the 3‐D similarity comparison requires 3‐D molecular structures (i.e., conformers) and that a molecule can have multiple conformers. Therefore, the 3‐D similarity between two molecules is assessed by computing 3‐D similarity scores for all possible conformer pairs arising from the combination of the conformers of the molecules, and selecting the highest score among them. For each compound in PubChem, a conformer model containing up to 500 diverse conformers is generated, among which up to 10 diverse conformers per compound are made accessible to the public and can also be used for 3‐D similarity evaluation in PubChem (Bolton et al., 2011; Bolton et al., 2011a; Kim et al., 2013).

### Critical Parameters and Troubleshooting

PubChem's search interface provides filters that allow users to refine hit records based on selected attributes. Each of the PubChem data collections has its own set of filters. For example, compound records can be filtered based on molecular properties (e.g., molecular weight, rotatable bond count, heavy atom count, hydrogen bond donor and acceptor counts, polar surface area, and XLogP) as well as the created date. The filters used on gene records include taxonomy groups (e.g., human, mouse, rat, etc.) and data source (e.g., BioAssay and Pathway). These filters help users to find information more specific to their needs.

Chemical structure searches in PubChem can be customized using various options available through the “Settings” button. It is worth mentioning that, because chemical structure searches are much more time‐consuming than text (keyword) searches, they are set by default to stop when a thousand hit compounds have been returned. While the search can be extended beyond this 1000‐hit limit (by checking the “Search All” box), only up to one million hits will be returned, at most. Therefore, a query structure should be specific enough not to exceed this limit.

The protocols in this article are designed to demonstrate the utility of PubChem, and can be readily modified and adopted for many other tasks. It is worth mentioning that these protocols are for interactive users who access PubChem data through web browsers (e.g., Google Chrome, Microsoft Edge, Safari, FireFox, etc.). When an interactive task needs to be repeated for a large number of PubChem records, it can likely be automated through PubChem's programmatic interfaces such as PUG‐REST (Kim, Thiessen, Bolton, & Bryant, 2015; Kim, Thiessen, Cheng, Yu, & Bolton, 2018) and PUG‐View (Kim et al., 2019). PubChem also supports the bulk download of its data through the PubChem FTP (file transfer protocol) site. Additional information about PubChem can be found in PubChemDocs (https://pubchemdocs.ncbi.nlm.nih.gov).

### Understanding Results

PubChem contains a massive amount of data, collected from hundreds of data sources. Although PubChem makes every effort to ensure high data quality, inconsistency may be found in the data from different sources. For this reason, PubChem preserves information on the provenance of data (i.e., what source the data originated from), so that users can go to the original data source and find additional information that may help them to understand the data contained in PubChem.

### Author Contributions

**Sunghwan Kim**: conceptualization, methodology, visualization, writing original draft, writing review and editing.

### Conflict of Interest

The authors declare no conflict of interest.

## Data Availability

All PubChem data, tools, and services are provided to the public free of charge.
